# First Report of Isolation and Molecular Characterization of Felid Herpesvirus-1 from Symptomatic Domestic Cats in Egypt

**DOI:** 10.3390/vetsci9020081

**Published:** 2022-02-15

**Authors:** Asmaa Magouz, Maha S. Lokman, Ashraf Albrakati, Ehab Kotb Elmahallawy

**Affiliations:** 1 Department of Virology, Faculty of Veterinary Medicine, Kafrelsheikh University, Kafrelsheikh 33516, Egypt; 2 Biology Department, College of Science and Humanities, Prince Sattam bin Abdul Aziz University, Alkharj 11942, Saudi Arabia; ms.hussein@psau.edu.sa; 3 Department of Zoology and Entomology, Faculty of Science, Helwan University, Cairo 11795, Egypt; 4 Department of Human Anatomy, College of Medicine, Taif University, P.O. Box 11099, Taif 21944, Saudi Arabia; a.albrakati@tu.edu.sa; 5 Department of Zoonoses, Faculty of Veterinary Medicine, Sohag University, Sohag 82524, Egypt

**Keywords:** feline herpesvirus 1, cats, molecular, CAM, Egypt

## Abstract

Feline herpesvirus 1 (FHV-1) is one of the main causes of upper respiratory tract infection in cats. Despite its veterinary importance, no previous studies investigated the occurrence of this virus in Egypt. In the present work, a total number of one hundred forty (*N* = 140) conjunctival and/or oropharyngeal swabs were collected from symptomatic cats during veterinary clinic visits located in two Egyptian provinces. Virus isolation was performed in the Chorioallantoic membranes (CAMs) of 12-days-old SPF eggs. Interestingly, the embryos showed stunting growth and abnormal feathering and infected CAMs showed edematous thickening and cloudiness with characteristic white opaque pock lesions. Polymerase chain reaction (PCR) amplification of the thymidine kinase gene (TK) was successful in 16/140 (11.4%) of the suspected cases. Two of the amplified genes were sequenced and the TK gene sequences of the FHV-1 isolates were highly similar to other reference strains in the GenBank database. Given the above information, the present study represents the first report of feline herpesvirus type 1 (FHV-1) in domestic cats in Egypt. Further studies on the causes of upper respiratory tract infections in cats as well as vaccine efficacy are needed.

## 1. Introduction

Feline rhinotracheitis is a contagious disease caused by feline herpesvirus type-1 (FHV-1) and is regarded as the main cause of conjunctival and corneal ulcers in cats, as well as upper respiratory tract infections [1]. FHV-1 was first isolated in the USA in 1957 from a cat suffering from an upper respiratory tract disease [2]. It is predicted that more than 90% of cats are seropositive to FHV-1, at least 80% of infected cats remain latently infected for lifetime, and after reactivation, approximately 45% of latently infected cats shed virus during their lives [3]. Clinical signs and severity vary depending on the virus strain, age, and immune condition of each animal, hence younger animals are more susceptible to infection [4]. Acute upper respiratory and ocular infection caused by feline herpesvirus often manifests as pyrexia, depression, anorexia, lethargy, serous to purulent ocular and/or nasal discharge, facial or nasal dermatitis, conjunctivitis, frequent sneezing, hyper salivation, coughing [5,6,7], ulcerative keratitis, anterior uveitis, eosinophilic conjunctivitis, keratoconjunctivitis sicca and corneal dendritic ulcers, which are pathognomonic [8,9]. Pneumonia and significant mortality rates have been observed in young kittens [10]. Rare forms of FHV-1-related skin ulcers, gingivostomatitis, gastritis, and pancreatitis have also been recorded [11,12]. A strong immune response can usually end the clinical disease within 2–3 weeks. However, it is predicted that 80% of infected cats will maintain latency in the trigeminal ganglia, and recurring episodes of rhinitis, conjunctivitis, and keratitis are prevalent upon reactivation from latency [13,14,15]. FHV-1 is a member of the order Herpesvirales, family Herpesviridae, subfamily Alphaherpesvirinae and genus Varicellovirus [16]. Virion size ranges from 120 to 180 nm, with a core carrying a double-stranded viral DNA genome, an icosahedral capsid which is surrounded by tegument layer, and a lipid envelope with glycoprotein spikes [16]. The viral DNA genome consists of unique long (UL) 104 Kbps and unique short (US) 30 Kbp sequences flanked by inverted repeat regions called terminal and inverted repeat long and terminal and inverted repeat short [9]. Antigenically, all FHV-1 isolates belong to the same serotype, and restriction enzyme analysis of their DNA reveals that they are generally homogeneous, although slight genetic changes have been documented for some strains [17]. FHV-1 has been shown to contain 23 virion associated proteins [18]. Four viral proteins have been considered as virulence factors: thymidine kinase (TK), serine/threonine protein kinase (US3), gE, and gC [19].

FHV-1 primarily infects domestic cats, but lions and cheetahs are also susceptible. Cats of all ages, sex or breeds are susceptible, but a severe syndrome is usually restricted to kittens of up to six months of age [20,21]. The natural routes of FHV-1transmission include the nasal, oral, and conjunctival pathways. Transmission occurs mostly through direct contact between infected and non-infected cats; however, indirect transmission can occur through contaminated cages, food and cleaning equipment [3]. FHV-1 is thought to be responsible for over 50% of all confirmed viral upper respiratory infections in cats [22]. Therefore, FHV-1 immunizations are included in the feline essential vaccines. The World Small Animal Veterinary Association strongly advises that kittens get vaccinated against FeHV-1 infection at 1–2 months, followed by a further dose after 2–4 weeks [23]. The FHV-1 vaccines used are usually combined with vaccines against feline panleukopenia virus and (FPV) feline calicivirus (FCV)[9]. Commercially accessible modified live vaccines and inactivated vaccines are usually safe and provide adequate protection, however they do not prevent infection, virus shedding, or the establishment of the carrier state [17,20,24]. A preliminary diagnosis of FHV-1 infection is usually made depending on the clinical signs. A case of FHV-1 can be suspected when pathognomonic dendritic ulcers are observed [25]. Clinically, there is a significant correlation between acute FHV-1 and FCV clinical features but FHV-1 infections are characterized by a high fever and corneal ulcers, however, ulcers of the palate, tongue, and throat are more common in FCV infections [26]. The direct fluorescent antibody (FA) test, enzyme-linked immune sorbent assay (ELISA), virus isolation (VI), and PCR are the routine laboratory procedures for demonstrating the presence of FHV-1 in tissue supernatants or swabs [16,27]. Although these approaches are adequate for detecting virus during the acute stages of infection, they may lack the sensitivity needed to detect FHV in chronic infections where viral shedding is low [1,28]. Reviewing the available literature, there is no available information about FHV-1 in Egypt. Vaccination with live attenuated vaccines is performed in Veterinary Clinics, although the percentage of the population that is really vaccinated is undetermined. To our knowledge, there has not yet been a described isolation of FHV-1 in Egypt. Given the above information, the aim of the present study was to report the isolation and molecular characterization of FHV-1 from symptomatic cats in the northern part of Egypt.

## 2. Materials and Methods

### 2.1. Ethical Considerations

The study was approved by the Research Ethics Committee of the Faculty of Veterinary Medicine, Kafrelsheikh University, and the institutional Review Board Number KFS-2021/06.

### 2.2. Sampling

**Figure 1:**
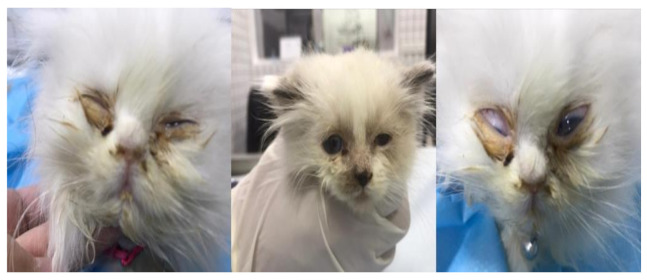
Nasal and ocular signs of cats infected with FHV-1.

One hundred forty conjunctival and/or oropharyngeal swabs were routinely sampled from symptomatic cats from during veterinary clinic visits located in two Egyptian provinces (Kafrelsheikh and Elgharbia) during September–December 2021. The cats were examined by licensed veterinarians and signs of upper respiratory tract disease (URTD) including fever, sneezing, coughing and serous to purulent ocular and/or nasal discharges were recorded. In addition, as shown in Figure 1, some ocular lesions including conjunctivitis and corneal ulcers in some cases were reported. The full details and data of the study cohort, reported clinical signs, and vaccination status of examined cats are shown in the Supplementary Materials (Table S1). Swabs were dipped in sterile tubes containing 1 mL phosphate-buffer saline (PBS) and shipped on ice to the Central diagnostic laboratory at the Faculty of Veterinary Medicine, Kafrelsheikh University where they were centrifuged at 13,000 rpm for 10 min at 4 °C, and the supernatants were stored at −80 °C.

### 2.3. Virus Isolation

Collected supernatants were treated with 100 IU/mL of penicillin and 100 μg/mL streptomycin stock solution and 0.2 mL were inoculated onto the CAMs of 12-days-old SPF eggs [29]. The eggs were incubated at 37 °C for 5–7 days and checked twice daily. Mortalities within the first 24 h of inoculation were considered non-specific. After three blind passages, the CAMs and fluid were harvested aseptically and cytopathic effect on CAM were recorded. CAMs containing pock lesions were homogenized with PBS, centrifuged, and the supernatants were kept at −20 °C until they were used in PCR.

### 2.4. DNA Extraction and PCR Amplification of Thymidine Kinase (TK) Gene

Viral genomic DNA was extracted from 300 μL of conjunctival and/or oropharyngeal swab suspensions using Gene Jet Viral DNA/RNA Extraction Kit (ThermoFisher Scientific, Waltham, MA 02451, USA) (Catalog No. K0821) as per producer’s protocol. DNA quality and concentrations were estimated by a NanoDrop (Quawell UV-Vis spectrophotometer Q5000). The purified DNA was stored at −20 °C till used. PCR assay was conducted in 25 μL volumes, consisting of 12.5 μL of 2X TOPsimple DyeMix-nTaq (enzynomics) (Yuseong-gu, Daejeon, Korea), 1 μL of forward and reverse primers, 3 μL of DNA and 7.5 μL of PCR grade water. PCR was conducted in an Applied biosystem 2720 thermal cycler using a set of primers, which are shown in Table 1, targeted amplification of a 293 bp of the TK gene [30]. Primers were synthesized by LGC Biosearch technology, Germany. The PCR cycle Protocol consisted of initial denaturation at 94 °C for 5 min, then 30 cycles of denaturation at 95 °C/30 s, annealing at 56 °C/30 s and extension at 70 °C/30 s with a final extension at 72 °C/5 min. For positive control, DNA was extracted from lyophilized Felocell 4 vaccine (Zoetis), while the negative control tube contained only PCR master mix and primers. Amplified PCR products were visualized using 2% agarose gel electrophoresis and the size of the PCR amplicons was determined using the 100 bp DNA marker (Thermo Fisher Scientific, Waltham, MA 02451, USA).

### 2.5. DNA Sequencing and Phylogenetic Analysis

Amplicons of FHV-1 TK gene of two selected isolates were purified using QIA quick PCR product extraction kit (Qiagen, Valencia, Spain) and transported to Macrogen Clinical Laboratory (Seoul, Korea) for DNA sequencing by the same primer sets using Big dye Terminator V3.1 cycle sequencing kit (Perkin-Elmer, Fostercity, CA, USA) in an Applied Biosystems 3130 genetic analyzer (ABI, Waltham, Massachusetts, USA). Sequence data were submitted to the GenBank databases under accession numbers OL840819 (FHV1Egypt/2021/1) and OL840820 (FHV1Egypt/2021/2). Blast analyses (BLASTn) (http://www.ncbi.nlm.nih.gov/, accessed on 10 January 2022) was performed to establish TK gene identity to GenBank accessions. The nucleotide sequences were aligned and compared with other FHV-1 reference strains available in the GenBank database by CLUSTAL W Multiple Sequence Alignment tool of MEGA X software. Phylogenetic analyses were conducted with the neighbour-joining method and the maximum composite likelihood model with 1000 bootstrap repeats using MEGA X software [31].

## 3. Results

### 3.1. Virus Isolation

**Figure 2:**
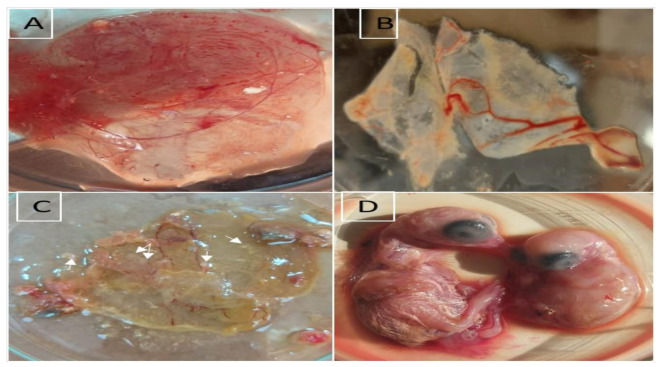
Virus isolation in chick CAM. (**A**) Normal non infected CAM; (**B**) Infected CAM showing cloudiness and thickening; (**C**) White pock lesions on infected CAM (arrows); (**D**) Left normal non infected 17-day-old embryo, Right infected 17-day-old embryo with stunted growth and abnormal feathering.

Embryos showed stunting growth and abnormal feathering and infected CAMs showed edematous thickening and cloudiness with characteristic white opaque pock lesions at the 5th–7th days post inoculation. The negative control sample showed no changes in the inoculated eggs (Figure 2).

### 3.2. Molecular Identification by PCR

Conjunctival and/or oropharyngeal swab suspensions and the infected CAMs of 16 samples (Mixed breeds and Persian peki breeds) were positive for the expected amplicon size of 293 bp corresponding to a portion of the TK gene.

### 3.3. Sequencing and Phylogenetic Analysis

**Figure 3:**
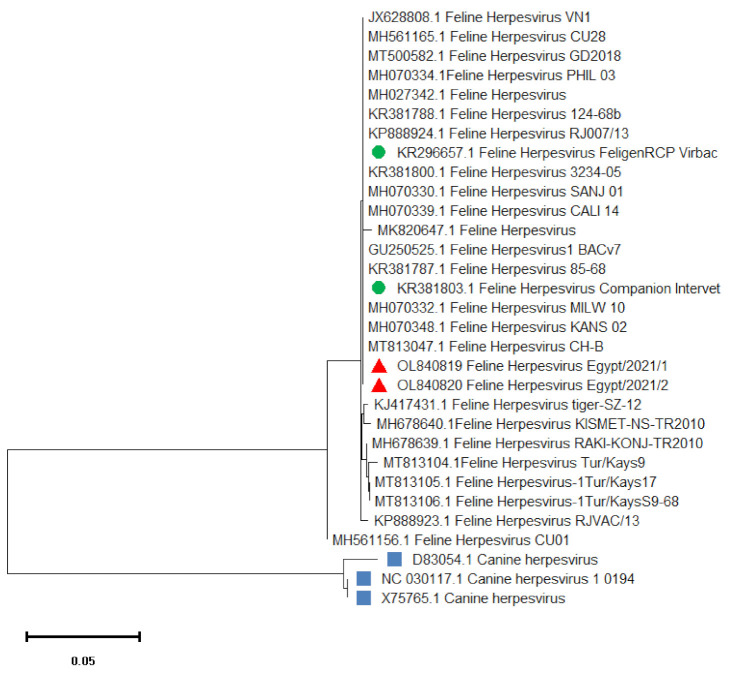
Phylogenetic tree based on the partial TK gene nucleotide sequences of FHV-1. The isolates of this study is indicated by red triangles. The tree was conducted with the neighbour-joining method and the maximum composite likelihood model with 1000 bootstrap repeats using MEGA X software.

Alignment and Phylogenetic analysis of the nucleotide sequence of the TK gene amplicon of the two isolates (FHV1Egypt/2021/1) and (FHV1Egypt/2021/2) revealed 99–100% sequence identity to most of FHV-1viruses isolated worldwide (Table 2). Our isolates showed 100% sequence homology with FHV-1 isolates FHV 1 KANS_02, FHV 1 PHIL_03, FHV 1 BACv7, FHV 1 VN1 and FHV 1 CH-B (GenBank accession numbers MH070348.1, MH070334.1, GU250525.1, JX628808.1 and MT813047.1) respectively. In addition, as shown in Figure 3, these isolates also showed 100% identity to the vaccinal strains FeligenRCP Virbac (KR296657.1) and Companion Intervet (KR381803.1).

## 4. Discussion

Feline upper respiratory tract infection is a common and serious concern for veterinarians and cat owners around the world. Among others, FHV-1 has been identified as one of the major causes of these infections [20]. In the present study, FHV-1 was isolated and identified from cats with clinical symptoms from two provinces in the northern part of Egypt. The overall PCR detection rate for FHV-1 DNA was low (11.4%). The present results are consistent with earlier published studies, as the detection rates of FHV-1 ranged from 9% to 88% [8,28,32,33,34,35], from 4% to 21% [25] and from 2.6–12% in privately owned cats [36]. This broad range might be attributed to the fact that detection rates differ depending on infection status, examined population, sample type, and detection technique [37]. In the present study, samples were collected from client-owned animals with varying clinical signs. In another investigation in which samples were collected from shelter cats, the prevalence rates were much higher (31%) [28] and (63%) [38]. It is also possible that the low incidence of FHV-1in cats in this study is attributable to their disease’s chronicity. FHV-1 was found in (1.9%) [39] and (33.3%) of cats with chronic conjunctivitis [40]. Most of the cats in this investigation showed clinical manifestations for more than two weeks prior to sample collection, suggesting that FHV-1 DNA detection was less common if disease was present for longer than one week [41]. Another possible reason is that the area where the samples were taken had a lower prevalence of FHV-1. The low detection rate of FHV-1 from cats with severe conjunctivitis in the study reported here was surprising. Taken into account, there are causes for respiratory and ocular diseases in felines other than FHV-1, including *Chlamydia felis*, *Bordetella* spp. and *Feline Calicivirus* (FCV) since conjunctivitis is not pathognomonic for any etiologic agent. There is high similarity between clinical symptoms of FHV-1 and FCV, but infections by FHV-1 are frequently characterized by a high fever and corneal ulcers, however, FCV is characterized by ulcers of the palate, tongue, and throat. Other agents, including *Chlamydia*, *Mycoplasma* and *Bordetella* may also resemble the clinical signs of FHV-1. However, it should be borne in mind that *Chlamydia* and *Mycoplasma* commonly cause primary conjunctivitis while *Bordetella* has been shown to cause acute bronchitis and pneumonia [26]. In addition, non-infectious causes of conjunctivitis might have similar clinical manifestations [20,28,37]. However, no attempt was made to detect the other possible causes of conjunctivitis. The conjunctival swab results are not as sensitive as those from conjunctival biopsies, which could explain some of the negative findings in this study [42]. Sample collecting techniques may also influence FHV-1 detection rates. In the present study, samples were collected by swabbing with a cotton swab, as this is the most common technique used in clinical practice. Although DNA was detected in 16 samples, it is possible that there were not sufficient cells containing FHV-1 DNA in the swab specimens. Investigators in other studies [43] have collected samples via conjunctival biopsy with an increased number of cells for testing, which thought to be related with a higher detection rate [25,37]. Recently, it was observed that DNA yields from dry swabs or brushes were higher than from fluid-suspended swabs [44]. Samples in this study were immersed in saline upon collection, which may have lowered DNA yields. Conversely, it was recorded in another investigation that there were no differences in the detection rates of FHV-1 DNA whether the samples were taken by swabs or biopsies, with the exception of samples from corneal sequestra where PCR results from cotton swabs were negative while those from biopsies were positive [45]. Overall, PCR-based molecular detection is mainly identified as a sensitive, specific, and fast method for detecting FHV-1 genomes [1].

Sequencing of the amplified DNA showed 99–100% identity with reference samples in the GenBank database while there was only 68–75% similarity in nucleotide sequences between Canine Herpesvirus. Notably, no significant differences were found between the nucleotide sequences of amplified TK gene derived from the current samples and previously published viral isolates. Overall, our findings show that FHV-1 genomes are highly conserved, which is consistent with prior findings that FHV-1 isolates are antigenically indistinguishable and have a high level of genomic identity, and that all FeHV-1 isolates antigenically belong to the same serotype [24]. The TK gene is a conserved gene, and the target fragment is located in a highly conserved region [30]. On the Contrary, other studies have found variations between FHV-1 isolates, such as differences in MluI cleavage patterns [46], rearrangements in the (gC) gene [47], and changes in the UL17 gene restriction endonuclease cleavage site [48].

For the isolation and identification of FeHV-1, feline cell cultures have commonly been used [49]. In this study FHV-1 were isolated on the chorioallantoic membrane of chick embryo which was similar to previous Herpesvirus studies including Bovine herpes virus-1 (BHV-1)which was isolated from bull semen by inoculation onto chorioallantoic membrane of SPF eggs [50], Gallid Herpes virus 1 of chicken [51], Equine herpesvirus 1 (EHV-1) [52], Herpes virus from a fisher (Martes pennanti) [53] and Herpes simplex virus [54]. Despite being vaccinated against feline herpesvirus-1, two of the cases in this investigation were positive for the virus. One probable explanation is that even though cats are FHV-vaccinated, they can become viral carriers and infect in-contact animals if challenged. Another possibility could be that kittens of persistently infected mothers can get infected and suffer from subclinical or mild infection prior to vaccination, when maternally acquired antibodies are reduced [10].Vaccinations may decrease the overall severity of the disease [20,55]. However, they do not prevent FeHV-1 infection, and as a consequence, the development of FeHV-1 latency [23]. FHV is prevalent in Egypt despite sporadic vaccination. Cats can encounter these viruses at a very young age and even vaccinated cats may become virus carriers and infect in-contact animals.

## 5. Conclusions

Given the above information, the presents study describes the first report of FHV-1 in domestic cats in Egypt which highlights the need for continued research into URT disease especially FHV-1 of domesticated cats. Our finding revealed the role of molecular methods in identification of these viruses. Further research is suggested for exploring the occurrence of the virus at large scale level.

## Figures and Tables

**Table 1:** Oligonucleotide primers used in the study.

Primer	Sequence	Target Gene	Amplified Product	Reference
FHV-F	GACGTGGTGAATTATCAGC	TK gene	293 bp	[30]
FHV-R	CAACTAGATTTCCACCAGGA	TK gene		

F = forward, R = reverse primer, TK = Thymidine Kinase.

**Table 2:** Alignment of the nucleotide sequences of thymidine kinase (TK) gene of FHV-1 with reference strains in GenBank database. GenBank accession numbers OL840819 (1); OL840820 (2); MT813106.1 (3); MH070348.1 (4); MH070334.1 (5); KR296657.1 (6); KR381803.1 (7); KP888923.1 (8); KJ417431.1 (9); GU250525.1 (10); JX628808.1 (11); MT813047.1 (12); NC_030117.1 (13); D83054.1 (14).

	% Diversity														
		1	2	3	4	5	6	7	8	9	10	11	12	13	14
% Identity	1-FHV 1 Egypt/2021/1	-	0	1	0	0	0	0	1	1	0	0	0	26	25
	2-FHV 1 Egypt/2021/2	100	-	1	0	0	0	0	1	1	0	0	0	26	25
	3-FHV 11Tur/KaysS9-68	99	99	-	1	1	1	1	1	1	1	1	1	32	31
	4-FHV 1 KANS_02	100	100	99	-	0	0	0	1	1	0	0	0	26	25
	5-FHV 1 PHIL_03	100	100	99	100	-	0	0	1	1	0	0	0	26	25
	6-FHV 1 FeligenRCP Virbac	100	100	99	100	100	-	0	1	1	0	0	0	26	25
	7-FHV1Companion Intervet	100	100	99	100	100	100	-	1	1	0	0	0	26	25
	8-FHV 1 RJVAC/13	99	99	99	99	99	99	99	-	1	1	1	1	26	25
	9-FHV 1 tiger-SZ-12	99	99	99	99	99	99	99	99	-	1	1	1	26	25
	10-FHV 1 BACv7	100	100	99	100	100	100	100	99	99	-	0	0	31	30
	11-FHV 1 VN1	100	100	99	100	100	100	100	99	99	100	-	0	26	25
	12-FHV 1 CH-B	100	100	99	100	100	100	100	99	99	100	100	-	26	25
	13-Canine herpesvirus 0194	74	74	68	74	74	74	74	74	74	69	74	74	-	2
	14-D83054.1Canine herpesvirus	75	75	69	75	75	75	75	75	75	70	75	75	98	-

## Data Availability

The data that support the findings of this study is s contained within the article.

## Supplementary Materials

The following are available online at https://www.mdpi.com/article/10.3390/vetsci9020081/s1. Table S1: The full details of the study cohort for each of the enrolled cat’s sex, age, breed, sample source, clinical signs, vaccination status and number of samples.
