# Supplementary material for: First Report of Isolation and Molecular Characterization of Felid Herpesvirus-1 from Symptomatic Domestic Cats in Egypt
Source: Vet Sci. 2022 Feb 15;9(2):81. doi: 10.3390/vetsci9020081 (PMC8874770; doi:10.3390/vetsci9020081)
Supplement: Supplementary file 1 [file vetsci-09-00081-s001.zip › vetsci-1566431-supplementary.pdf]

veterinary sciences

MDPI

Supporting Material

First report of Isolation and Molecular Characterization of Felid Herpesvirus-1 from Symptomatic Domestic Cats in Egypt

Asmaa Magouz [1*], Maha S. Lokman [2,3], Ashraf Albrakati [3], and Ehab Kotb Elmahallawy [4*]

Table S1. The full details of the study cohort for each of the enrolled cat's sex, age, breed, sample source, clinical signs, vaccination status and number of samples.

Breed	Age (months)	Sex	Sample source	Clinical signs	Vaccination status	Number of samples
Persian himalaya	6	Female	Conjunctival swab	Conjunctivitis	vaccinated	4
Persian himalaya	5	Female	Oropharyngeal swab	URTD	vaccinated	4
Persian (moon face)	9	Female	Oropharyngeal swab	URTD	vaccinated	4
Persian (moon face)	3	Male	Conjunctival swab	Corneal ulcer	unknown	4
Mix breed	12	Male	Conjunctival swab	Conjunctivitis	vaccinated	4
Mix breed	6	Female	Oropharyngeal swab	URTD	vaccinated	4
Mix breed	4	Male	Oropharyngeal swab	URTD	vaccinated	4
Persian (moon face)	6	Male	Oropharyngeal swab	URTD	vaccinated	4
Mix breed	3	Female	Oropharyngeal swab	URTD	Unknown	4
Mix breed	9	Male	Conjunctival swab	Conjunctivitis	vaccinated	4
Persian himalaya	2	Female	Oropharyngeal swab	URTD	Not vaccinated	4
Persian (moon face)	15	Male	Oropharyngeal swab	URTD	vaccinated	4
Mix breed	7	Female	Conjunctival swab	Corneal ulcer	vaccinated	4
Persian himalaya	3	Female	Oropharyngeal swab	URTD	vaccinated	4
Mix breed	3	Female	Conjunctival swab	Conjunctivitis	Not vaccinated	4
Mix breed	2	Male	Oropharyngeal swab	URTD	vaccinated	4
Mix breed	4	Female	Oropharyngeal swab	URTD	vaccinated	4
Persian (moon face)	3	Female	Conjunctival swab	Conjunctivitis	vaccinated	4

Mix breed	12	Male	Oropharyngeal swab	Corneal ulcer	vaccinated	4
Persian himalaya	18	Female	Conjunctival swab	Conjunctivitis	Unknown	4
Persian peki	6	Male	Oropharyngeal swab	URTD	vaccinated	4
Mix breed	5	Female	Oropharyngeal swab	URTD	vaccinated	4
Mix breed	9	Female	Conjunctival swab	Corneal ulcer	vaccinated	4
Persian peki	3	Male	Oropharyngeal swab	URTD	Not vaccinated	4
Persian himalaya	4	Male	Conjunctival swab	Conjunctivitis	vaccinated	4
Persian himalaya	4	Female	Conjunctival swab	Conjunctivitis	vaccinated	4
Persian peki	15	Male	Oropharyngeal swab	URTD	vaccinated	4
Mix breed	2	Male	Oropharyngeal swab	URTD	Not vaccinated	4
Mix breed	18	Male	Oropharyngeal swab	URTD	vaccinated	4
Persian peki	2	Female	Oropharyngeal swab	URTD	Not vaccinated	4
Mix breed	3	Male	Oropharyngeal swab	URTD	vaccinated	4
Persian peki	12	Male	Conjunctival swab	Conjunctivitis	unknown	4
Persian (moon face)	4	Female	Conjunctival swab	Corneal ulcer	vaccinated	4
Mix breed	6	Male	Conjunctival swab	Conjunctivitis	vaccinated	4
Persian himalaya	7	Male	Conjunctival swab	Conjunctivitis	vaccinated	4
